# Accretionary prism collapse: a new hypothesis on the source of the 1771 giant tsunami in the Ryukyu Arc, SW Japan

**DOI:** 10.1038/s41598-018-31956-8

**Published:** 2018-09-11

**Authors:** Yukinobu Okamura, Azusa Nishizawa, Yushiro Fujii, Hideaki Yanagisawa

**Affiliations:** 1 Research Institute of Earthquake and Volcano Geology, National Institute of Advanced Industrial Science and Technology (AIST), 1-1-1 Higashi, Tsukuba, Ibaraki 305-8567 Japan; 2 Hydrographic and Oceanographic Department, Japan Coast Guard, 3-1-1 Kasumigaseki, Chiyoda-ku, Tokyo 100-8932 Japan; 3 National Research Institute for Earth Science and Disaster Resilience, 3-1 Tennodai, Tsukuba, Ibaraki 305-0006 Japan; 4 International Institute of Seismology and Earthquake Engineering, Building Research Institute, 1 Tachihara, Tsukuba, Ibaraki 305-0802 Japan; 5 Department of Regional Design, Faculty of Liberal Art, Tohoku Gakuin University, 2-1-1 Tenjinzawa, Izumi-ku, Sendai, Miyagi 981-3193 Japan

## Abstract

The giant 1771 Yaeyama tsunami occurred in the southwestern part of the Ryukyu Arc, a region on an obliquely subducting plate boundary, which shows no direct evidence of inter-plate coupling. Studies of tsunami boulders and deposits suggest that the recurrence interval of comparably giant tsunamis is roughly 500 to 1000 years. Tsunami source models, which include either slip on a shallow plate boundary or active faulting plus a landslide on the overriding plate, are controversial because of inconsistencies in the geophysical and geological data. We discovered a seafloor depression that is approximately 30 km wide and 80 km long extending in the ESE-WNW direction. This depression is accompanied by a seaward bulge on the accretionary prism along the Ryukyu Trench, which is based on detailed bathymetric data and interpreted to be the result of accretionary prism collapse and seaward displacement by rotational slide. A simple tsunami simulation shows that the slide is a plausible source of the 1771 tsunami. We propose a collapse model, in which the accretionary prism remained over-steepened as strike-slip faulting removed the prism toe. Our model indicates that some oblique subduction zones are capable of generating giant tsunamis regardless of weak or strong coupling.

## Introduction

Giant tsunamis, one of the most devastating natural hazards in the world, are typically caused by earthquakes that release large amounts of accumulated elastic strain along a subducting plate boundary that has been coupled for several hundred years^1–5^. Recent observations of crustal movement and seismicity have resulted in detailed documentation of strain accumulation along subducting plate boundaries^6–8^, which has led to expectations that the locations and magnitudes of future giant tsunamis can be evaluated. However, the 1771 Yaeyama giant tsunami resulted in runups of 30 m and approximately 12,000 deaths in the Sakishima Islands in the southwestern Ryukyu (Nansei Shoto) Arc^9–11^, where direct evidence of plate boundary coupling has not been observed^6,12,13^. Satellite geodetic measurements on the islands along the arc show southeast to southward motion, which is attributed to substantial slip along the plate boundary and the opening of the Okinawa Trough^13^. These measurements have also detected slow slip events along the plate boundary at depths of 0 to 60 km^14,15^. Low- and very low-frequency earthquakes have also been detected along this part of the plate boundary at depths of 0 to 60 km^16,17^. These geophysical observations along the Ryukyu Arc suggest that the typical locked zone is absent; however, GPS measurements from Okinawa Island, approximately 110 km from the trench, cannot detect inter-plate coupling of a seaward zone approximately 30 km wide along the trench^18^. A density structural model^19^ of the Ryukyu Arc also suggested strong coupling along the plate boundary. Thus, the possibility of inter-plate coupling along the shallowest part of the plate boundary has not been excluded^11,18,19^, and the question as to whether the shallow plate boundary is coupled remains controversial.

**Figure 1:**
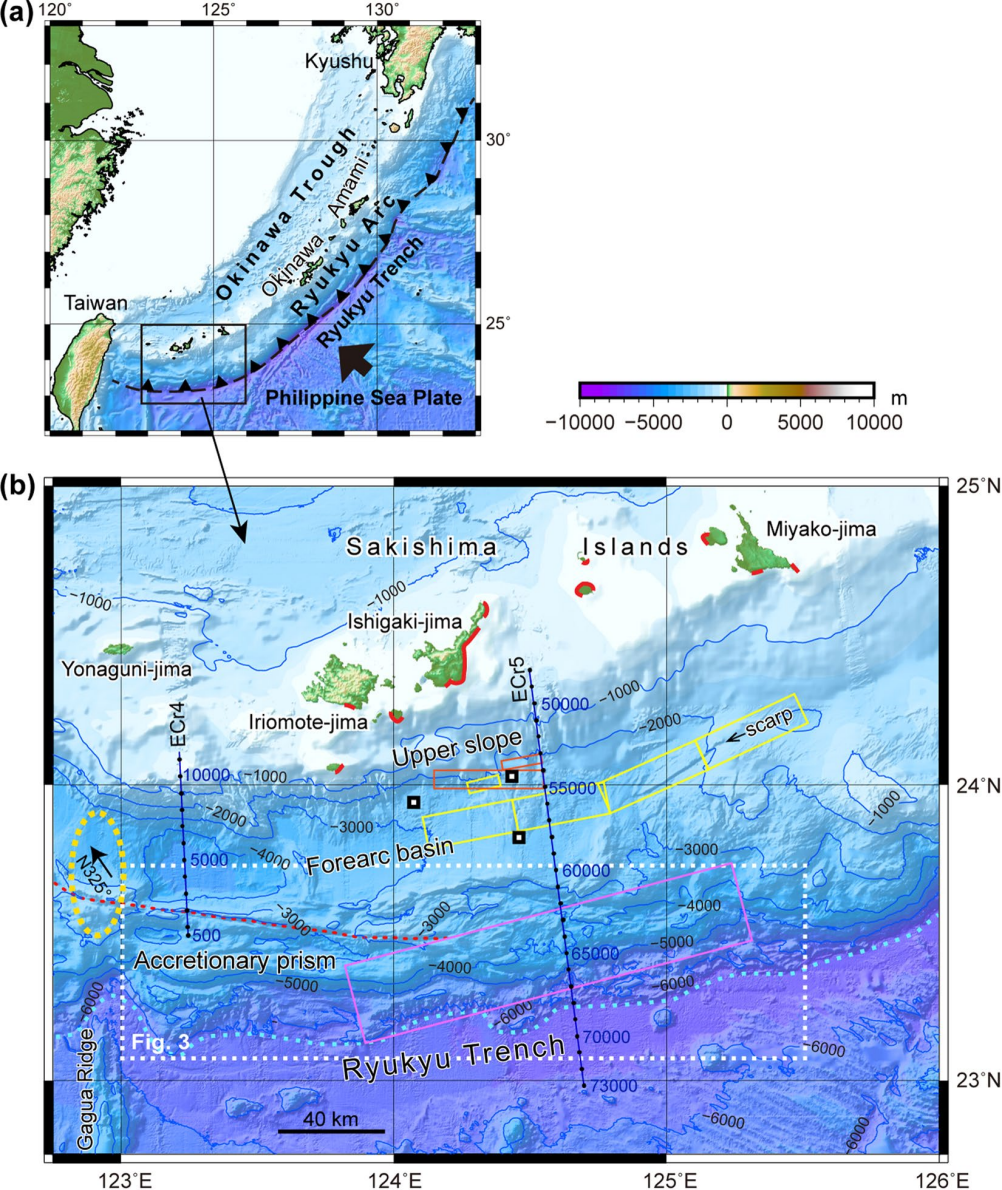
Location and bathymetry of the southwestern Ryukyu Arc. (**a**) Index map of the Ryukyu Arc. (**b**) Bathymetry of the southwestern Ryukyu Arc. Coasts marked in red contain tsunami deposits and boulders^9,10^. Previously proposed source models of the 1771 tsunami are indicated by orange^23,24^, yellow^25^ and pink^26^ rectangles. Blue lines indicate the seismic survey lines with CDP numbers. Small squares indicate the locations of sediment cores^43^. The orange dotted line is a strike-slip fault and the sky blue dotted lines indicate the deformation front. The yellow dashed oval shows an uplift zone in the accretionary prism and the forearc basin, and the arrow in the oval indicates the azimuth of the relative motion between Yonaguni-jima and the PSP^31^. The maps were produced using GMT 4.5^46^ and Adobe Illustrator CS6.

The 1771 tsunami was high in the eastern part of the Sakishima Islands (Fig. 1)^9,10^. Historical documents and tsunami deposits, including displaced “tsunami boulders,” indicate that the 1771 tsunami had runups of approximately 30 m along the southern and eastern coasts of Ishigaki-jima and ones greater than 10 m along the western coast of Miyako-jima and small neighbouring islands. The opposite coasts of Ishigaki-jima and Miyako-jima were damaged to a small degree by the tsunami. Age data from the tsunami deposits and boulders suggested that comparable tsunamis have occurred at intervals of roughly 500 to 1000 years^9–11,20^. The distribution of tsunami boulders is restricted to the Sakishaima Islands, and no tsunami boulders during the last 2000–3000 years have been found in the northern and middle parts of the Ryukyu Arc^21,22^.

Based on these ancient tsunami records, source models that reproduce the 1771 tsunami height have been proposed in the forearc area between 123°45′ E and 125°30′ E, south of Ishigaki-jima and Miyako-jima (Fig. 1b). Some of the models^23–25^ have attributed the tsunami to active reverse faults plus landslides on the upper slope and forearc basin. Another simple model^26^ assumed a slip on the plate boundary along the trench under the accretionary prism, which was attributed to a tsunami earthquake. Splay faults branching from the plate boundary, which were interpreted from seismic profiles, have also been proposed as possible sources of the tsunami^17,27^.

The forearc slope of the southwestern Ryukyu Arc is composed of a steep upper slope, forearc basin and accretionary prism from north to south (Fig. 1b). These forearc structures west of 122°40′ E have been disrupted due to tectonic movements related to the arc-continent collision along Taiwan, back-arc rifting of the Okinawa Trough and high subduction obliquity of the Philippine Sea Plate (PSP), which carries ridges and seamounts^28–30^. The forearc slope east of 123°E trends in the E-W to ENE-SWS direction and is less deformed. The accretionary prism comprising a topographic ridge decreases in size to the east because of the decrease in sediment thickness in the Ryukyu Trench. The prism has been sliding to the west relative to the arc along dextral strike-slip faults, which are clearly imaged by bathymetric map along the landward prism margin (Fig. 1b), and the fault motion is interpreted to have been caused by slip-partitioning of the oblique subduction of the PSP^29,30^.

The amount of accretionary prism dislocation along the strike-slip fault was estimated from the prism deformation geometry, which was caused by N-S trending Gagua Ridge subduction at 123°E^29,30^. The accretionary prism was indented by the ridge subduction, and the forearc basin basement was uplifted at the northern ridge extension (Fig. 1b), which suggests that the ridge subducted approximately 70 km from the trench. Because the relative motion between the PSP and Yonaguni-jima at the southwestern end of the Ryukyu Arc is estimated to be N325° at approximately 10.7 cm/y^31^ (Fig. 1b), the ridge under the forearc basin is inferred to have begun subducting from the trench approximately 50 km east of the present ridge position approximately 800,000 years ago. If the accretionary prism was fixed to the Ryukyu Arc, the ridge should have swept the accretionary prism and left a 50 km wide indentation on the prism. However, the bathymetric map shows that the width of the indentation east of the ridge is approximately 10 km, which is attributed to westward dislocation of the accretionary prism of approximately 40 km along the strike-slip fault over the last 800,000 years.

In this paper, we propose that the tsunami was generated by a large-scale collapse and a slide of the accretionary prism based on detailed bathymetric data, and seismic reflection and refraction data from the forearc slope of the SW Ryukyu Arc between 123°E and 125°10′E. We also present our hypothesis that the collapse was caused by a large displacement along the dextral strike-slip fault, which obliquely truncated the accretionary prism. Our model explains how giant tsunamis can be generated also along weakly coupled subduction zones. Besides, our results are important for the evaluation of future tsunamis affecting the Ryukyu Arc Islands.

## Results

### Upper slope and forearc basin

**Figure 2:**
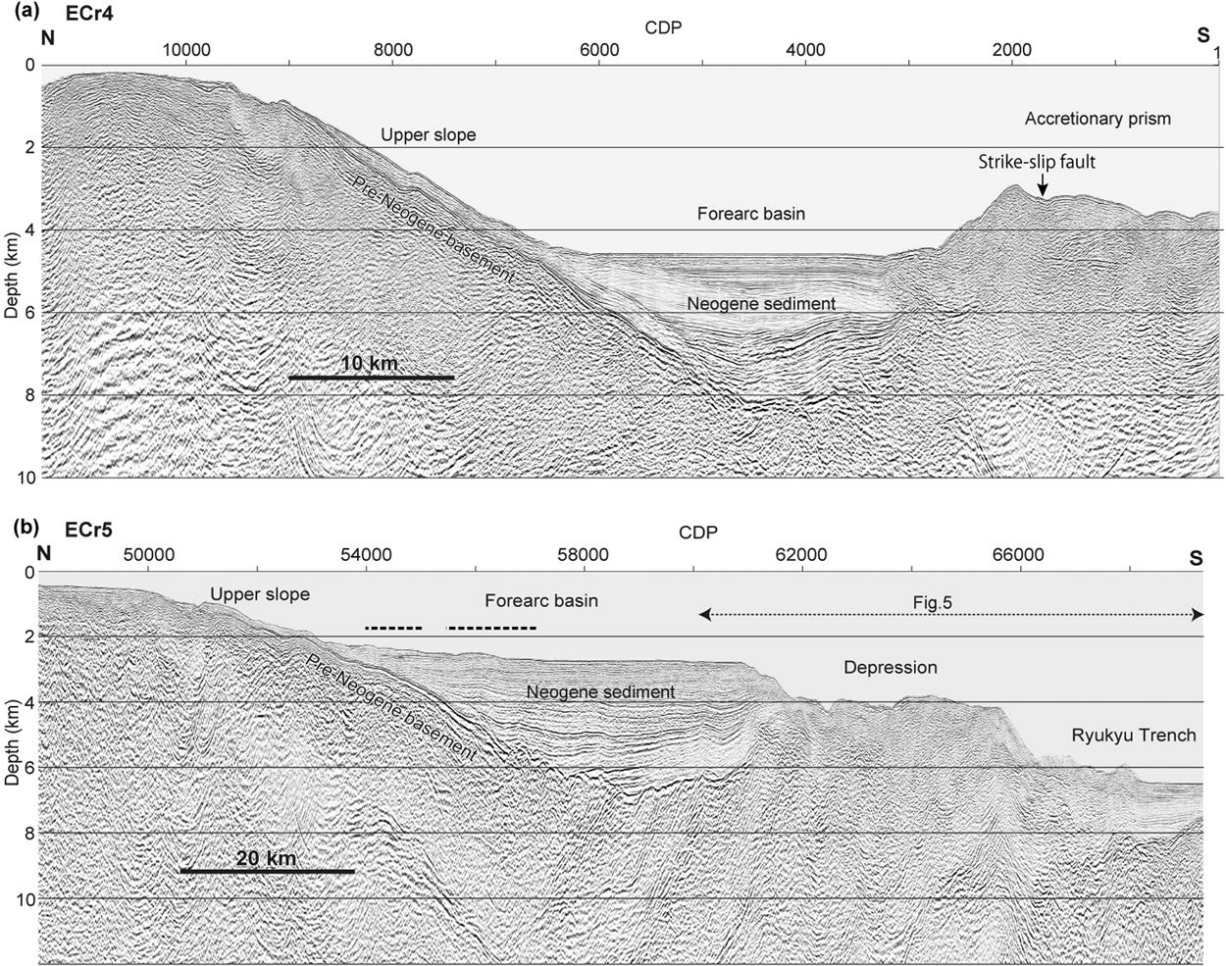
Seismic profiles of forearc slopes seaward of the Sakishima Islands. (**a**) Seismic profile of ECr4, which shows the topographic high bounding the seaward margin of the accretionary prism. (**b**) Seismic profile of ECr5, in which the seaward topographic high is absent. Approximate locations of the fault models^23,25^ for the 1771 tsunami source are shown by dashed lines above the forearc basin. Locations of the profiles are shown in Fig. 1. The image was produced using SPW 2.2 [http://www.parallelgeo.com/] and Adobe Illustrator CS6.

Two seismic profiles (ECr4 and ECr5) obtained in the study area show that the upper slope and forearc basin between 123°E and 125°E consist of a pre-Miocene basement overlain by Neogene sedimentary sequences, which increase in thickness from a few hundred metres under the upper slope to more than 3000 m in the forearc basin^32^ (Fig. 2). The upper slope has been incised by many canyons and gullies, but there are no apparent large-scale slope failures (Fig. 1b). The forearc basin is divided into several sub-basins, which are bounded by gentle uplift zones^29,30^. Sediments in the basin gently undulate at depth but are horizontal in the uppermost 1 km (Fig. 2). No active reverse faults and hanging-wall anticlines are apparent in the upper slope or forearc basin based on these profiles, although a linear scarp approximately 70 km long has been mapped in the basin east of 124°55′ E (Fig. 1b).

On seismic profile ECr 5 (Fig. 2b), the upper 1 km of sediments in the forearc basin at approximately 2700 m bsl are horizontally bedded (CDP 54000–61000 in Fig. 2b) and truncated by scarps at the seaward margin (CDP 61000–62000 in Fig. 2b). Horizontal successions up to 1000 m thick are not naturally deposited without being confined by a seaward topographic high, which is the case in seismic profile ECr4 (Fig. 2a), and we hypothesize that the horizontal sediments were trapped by a seaward topographic high that has been removed.

Landward-dipping reflections are observed in the forearc basin basement (CDP 58000–62000 in Fig. 2b). These reflections are similar to a reflection that was interpreted as a splay fault^17^; however, the parallel reflections are truncated by an unconformity under the forearc basin and covered with Neogene sediments. We interpreted the landward-dipping reflections to be structures in pre-Neogene rocks.

### Accretionary prism

**Figure 3:**
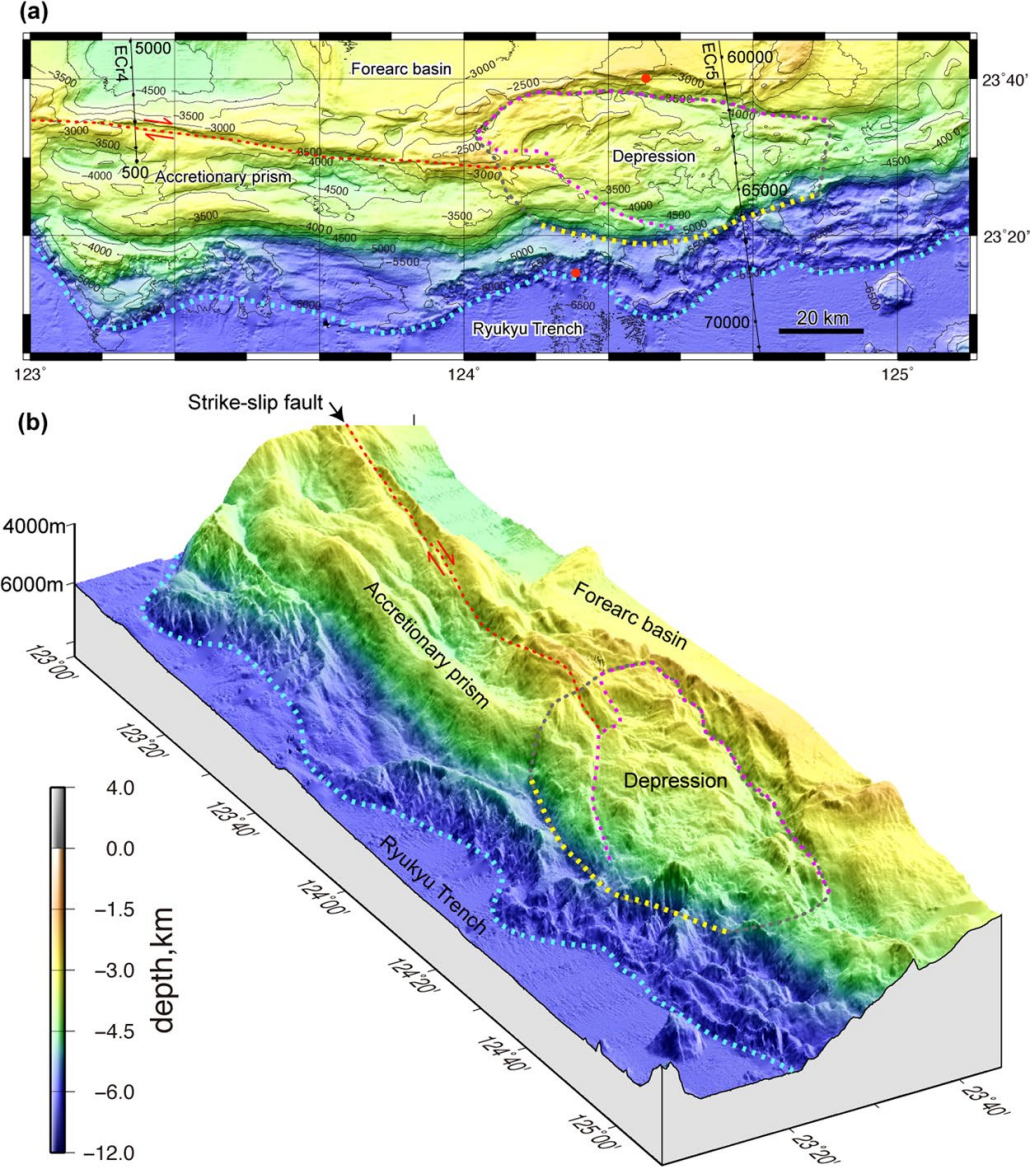
Detailed topography of the accretionary prism south of the Sakishima Islands. (**a**) Plain map and (**b**) bird’s-eye view of the accretionary prism show two major ridges and a strike-slip fault (orange dotted line) west of 124°E and a depression east of 124°E (pink dotted line). The seaward bulge and deformation front are indicated by yellow and sky blue dotted lines. The inferred slide mass is outlined by the grey dotted line. Dive survey sites^36^ where slope failures were observed are indicated by red circles. The map area is shown in Fig. 1b. The images were produced using GMT 4.5^46^ and Adobe Illustrator CS6.

The accretionary prism forms a topographic ridge approximately 30 km wide and comprises two large continuous ridges at 2500–3500 m bsl, which are parallel to the trench between 123°E and 124°E and smaller ridges below 5500 m bsl seaward of these major ridges mark the deformation front (Figs 1b and 3). The dextral strike-slip fault trending ESE obliquely cuts the major landward ridge on the accretionary prism (Figs 1b and 3).

**Figure 4:**
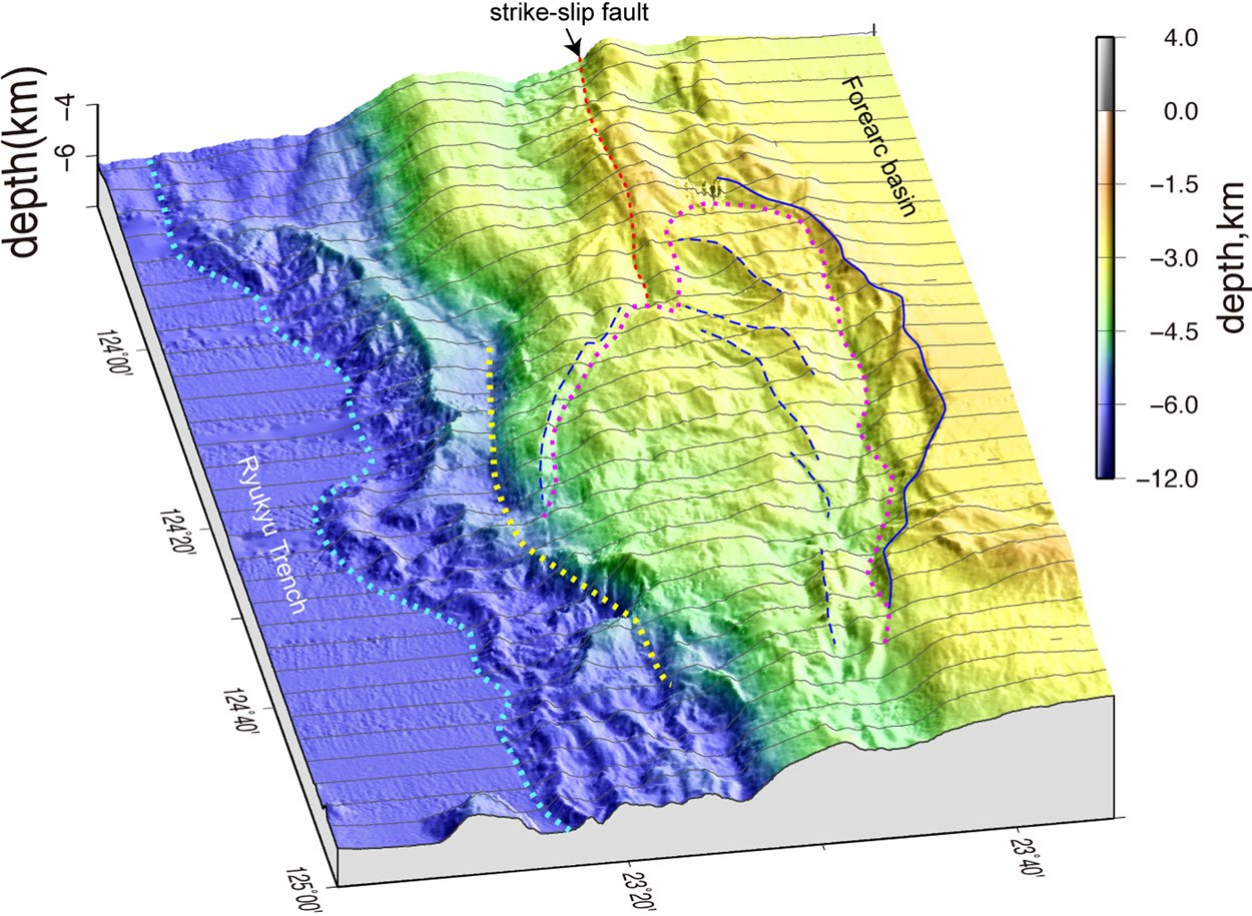
A bird’s-eye view of the shaded seafloor map. The seafloor bathymetric profiles at 2.5′ longitude intervals are indicated by grey lines to emphasize the depression geometry. The solid blue line indicates the upslope margin of the landward scarps, and the broken blue lines are the minor scarps in the depression, which are outlined by a pink dotted line. The yellow and sky blue dotted lines indicate the seaward bulge and deformation front south of the depression. The image was produced using GMT 4.5^46^ and Adobe Illustrator CS6.

The accretionary prism topographic high, which includes the major ridges and strike-slip fault, disappear east of 124°E because of an approximately 80 km long and 30 km wide depression that extends in the ESE-WNW direction (Figs 3 and 4). The landward depression margin consists of overlapping and concave-downward scarps, which are aligned along a concave-seaward curved line for approximately 80 km. The total relief of the landward margin is higher than 1000 m (Fig. 3a). The gently undulating floor of the depression descends towards the east from 3000 to 4500 m bsl, and minor scarps that trend NE-SW to E-W are apparent on the landward side of the depression. The steep slope to the seaward depression margin, which is expressed by depth contours between 4500 and 5000 m bsl, forms a seaward bulge that is approximately 70 km wide and between 124°10′E and 124°50′E (Figs 3 and 4). The western part of the bulge has a smooth, ESE to E-W trending slope that continues from the seaward slope of the accretionary prism to the west of 124°E, which indicates that the slope has not been deformed. The boundary between the slope and the depression is a north-down continuous scarp. The eastern part of the bulge strikes ENE and is highly disrupted by slope failures. The deeper ridges along the deformation front between 6000 and 6500 m bsl also bulge seaward between 124°10′ and 124°45′E, which is sub-parallel to the landward slope bulge.

**Figure 5:**
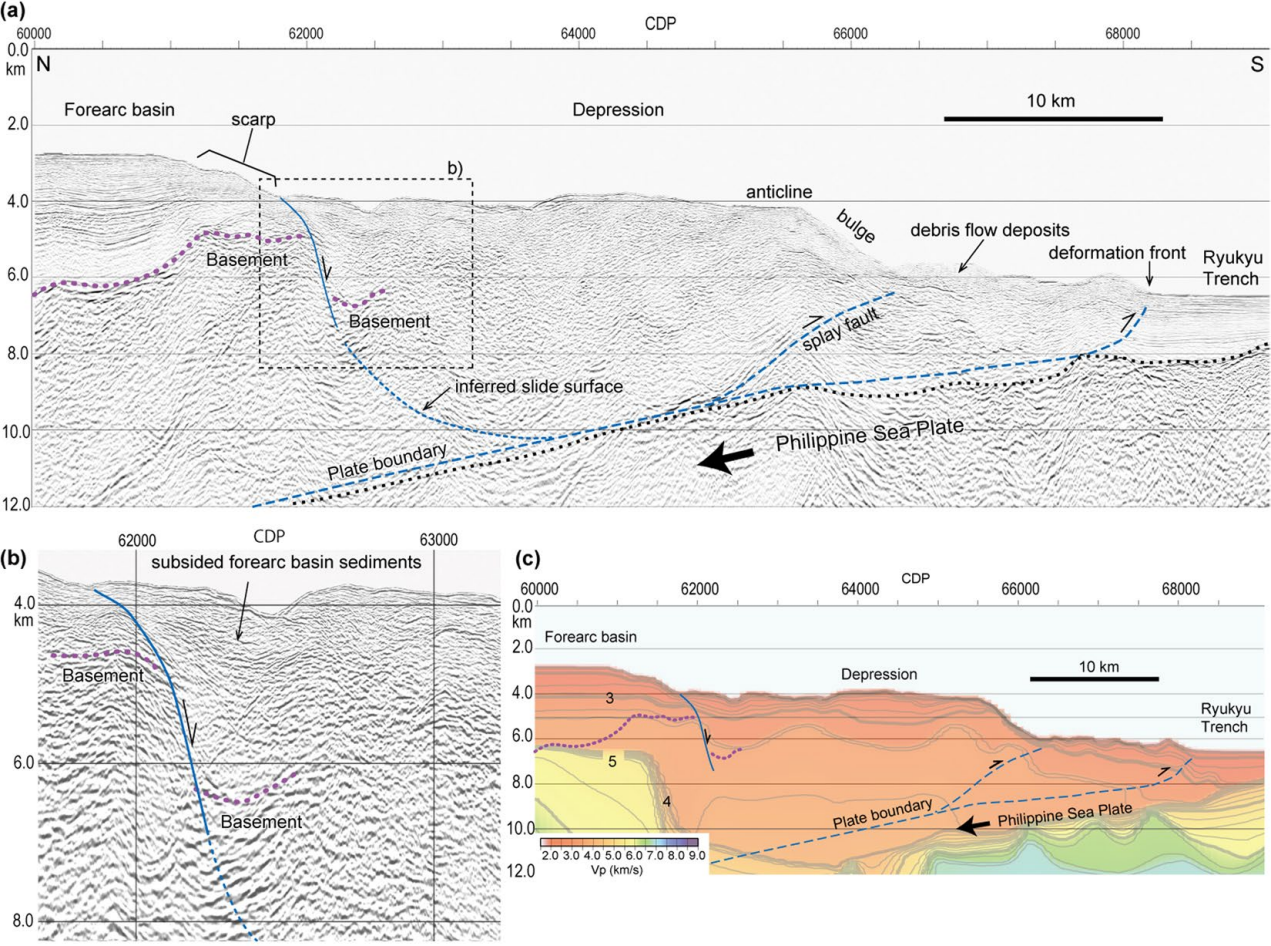
(**a**) Seismic cross section of the accretionary wedge along survey line ECr5. (**b**) Close up structure of the landward depression margin. (**c**) Velocity section of survey line ECr5, which is the same area as section a. Purple dotted lines are basement reflections. The plate boundary is based on the reflection profile. The images were generated with SPW 2.2 [http://www.parallelgeo.com/], GMT 4.5^46^ and Adobe Illustrator CS6.

Seismic profile ECr5 clearly shows the disappearance of the accretionary prism topographic high (Fig. 2b). The landward marginal part of the depression is underlain by sedimentary sequences that are approximately 2 km thick and bounded by a strong, basal reflection (CDP 62000–62500 in Fig. 5a,b), which can be correlated with the sediments and basement of the forearc basin. The abrupt change in the basement depth from 4.5 km under the forearc basin to 6.5 km under the depression suggests that the landward edge of the depression has been downthrown by approximately 2 km. In the velocity structure (Fig. 5c), a southward 1 to 2 km-layer deepening with a velocity of 3.2 to 3.5 km/s also supports the depression subsidence hypothesis. These reflections disappear seaward of CDP 62500 (Fig. 5a,b), which suggests that the depression is highly deformed. Farther seaward between CDP 64500 and 66000 (Fig. 5a), parallel reflections comprising an anticline are observed from just below the seafloor to a depth of 8 km bsl, which is interpreted as a hanging wall anticline above a splay fault that is branched from the detachment fault along the plate boundary (Fig. 5a). No large debris flow deposits are observed seaward of the depression (Figs 2 and 3).

### Collapse of the accretionary prism

The pairing of the depression and 70 to 80-km-wide seaward bulge on the accretionary prism (Fig. 4) is explained as the collapse of the accretionary prism and subsequent sliding seaward. The simple convex landward and seaward geometry of the margins suggest that the entire depression behaves as a single slide block, which is bounded by a continuous slide surface at its base, because multiple slide blocks would show multiple convex geometries along the landward and seaward margins. The south downthrown slide surface along the landward depression edge can be traced to depths greater than 7 km, and forearc sediments south of the slide surface have subsided without large deformation. The seaward bulge consists of an anticline above the splay fault, implying that the bulge slid seaward along the basal detachment and splay fault, which preserved its structure (Fig. 5a). The lack of extensive debris flow deposits covering the slope and trench seaward of the depression indicates that it is unlikely that the accretionary prism topographic high was removed by shallow slope failure. We interpreted the depression as a large-scale slide mass of approximately 80-km-wide moving towards the trench above a deep slide surface, which extended to the plate boundary at approximately 10 km bsl (Fig. 5a) and is similar to a rotational slide^33^. The depression area is approximately 1800 km^2^ and that of the slide mass, which includes the seaward bulge (Fig. 3) is approximately 2250 km^2^.

A strike-slip fault is clear on the accretionary prism west of 124°E and is suddenly obscured in the depression (Fig. 3), but the lateral displacement must have occurred somewhere in the depression. An indistinct trace of strike-slip fault has been inferred in the depression^34^, and the landward margin was interpreted to be a strike-slip fault^27^. A similar strike-slip fault in the accretionary prism has been reported along the Nankai Trough^35^. The fault accompanies a depression and flower structure, but the structural modification of the accretionary prism due to the fault is limited to a narrow zone of less than 5 km in width. Considering the structure of the accretionary prism along the Nankai Trough, we infer that strike-slip faulting cannot generate a large-scale depression on the accretionary prism between 124°10′E and 124°50′E. We will discuss the role of the strike-slip movement in the accretionary prism later in this manuscript.

Parts of the depression and deeper flat terrace between CDP 66500 to 67500 are covered with a thin layer of poorly reflecting sediments, which suggests that the seafloor is covered with slump and debris flow deposits (Fig. 5a). Observations from a remotely operated vehicle and manned submersible have documented slope failures on the seafloor along the landward scarp and on the ridge along the deformation front^36^ (Fig. 1b). These results indicate that the seafloor in and around the depression is unstable and that shallow slope failure is a significant process, but the large-scale geometry and structure described above cannot be explained by surface slumps and slides.

### Tsunami source

Previously proposed models have placed the source of the 1771 tsunami on the shallow upper slope^23–25^ or the toe of the forearc slope^26^, and all previous studies have approximately reproduced the heights of the tsunami. These results suggest that a tsunami simulation cannot provide a strong constraint on the tsunami source. In our model, a collapse of the accretionary prism at the forearc slope toe approximately overlaps the plate boundary model^26^, and thus, the slide is expected to be able to generate tsunamis comparable to the 1771 tsunami.

**Figure 6:**
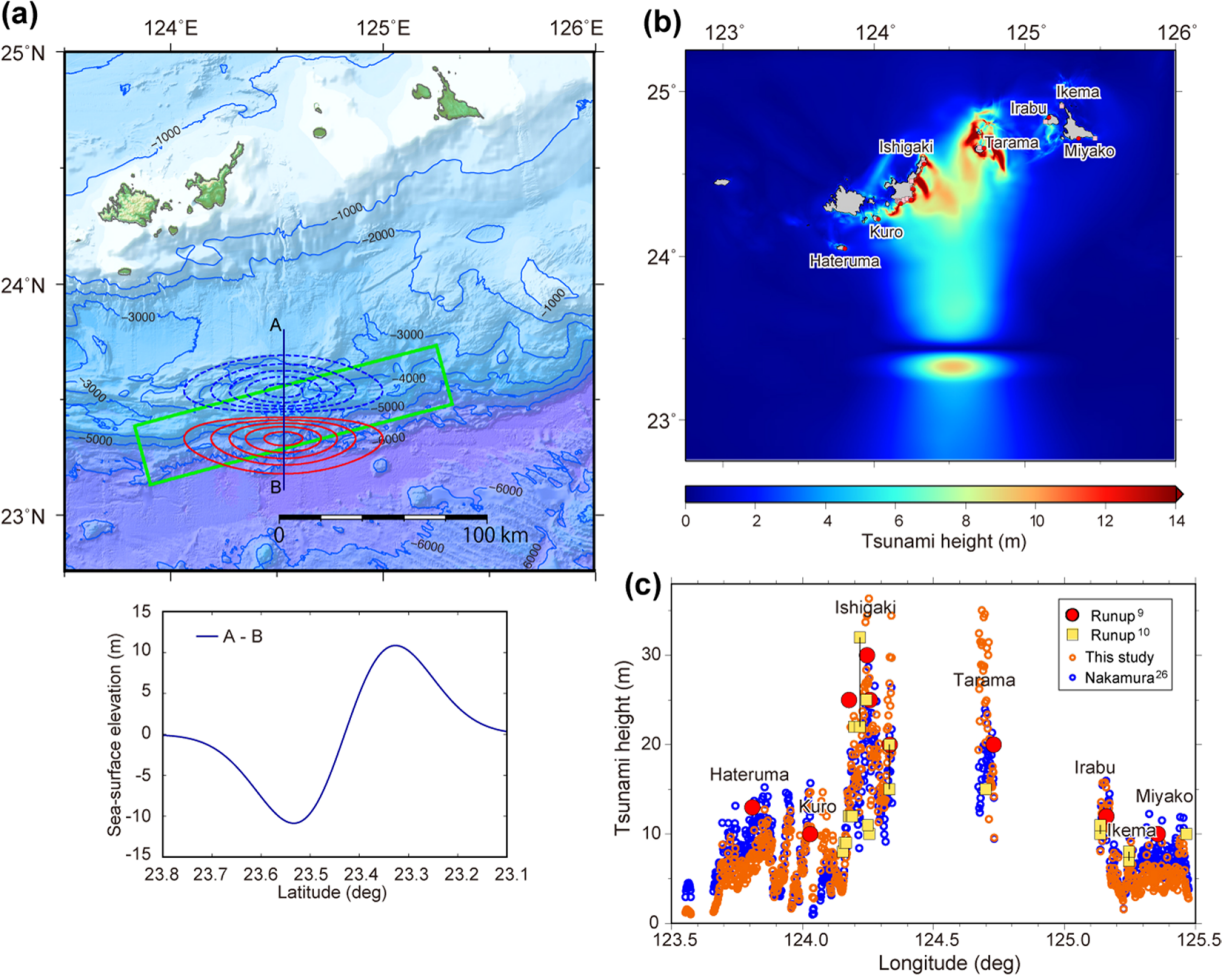
Estimation of tsunami height using the landslide model. (**a**) Map view of the sea-surface elevation resulting from the slide model. Contours indicate the sea-surface uplift (red) and subsidence (blue) at 2 m intervals. The fault model on the plate boundary^26^, which has a width of 30 km, is also indicated by the green rectangle. The profile of sea-surface elevation along line A-B is shown under the map. (**b**) Map view of calculated maximum tsunami heights. (**c**) Tsunami height along the coasts of the Sakishima Islands. Pink and blue open circles indicate tsunami heights computed from this model and Nakamura^26^ respectively. Large red circles and yellow squares indicate the estimated runup heights of the 1771 tsunami^9,10^. All figures were produced using GMT 4.5^46^ and Adobe Illustrator CS6.

We estimated the height of the tsunami generated by such a slide mass by modelling the accompanying sea-surface elevation as an approximate initial 3D tsunami source^37,38^. Broader discussions on landslide tsunami mechanisms and modelling techniques are introduced in review papers^39,40^. A rotational landslide would cause subsidence in the landward part and uplift in the seaward part of the slide mass^41^. In this study, to provide an initial tsunami source which is similar to that caused by the slump, we adopted predictive equations (Eq. (14) with Eqs. (4) and (9))^38^ with a failure length (b) of 30 km and failure width (w) of 80 km corresponding to the depression area (Fig. 6a). The failure thickness (T), slope inclination (θ) and slump motion distance (S) were set to 6 km, 6° and 450 m, respectively, which approximately correspond to the inferred slide surface shown in Fig. 5a. The other parameters of specific density (${\rm{\gamma }}$), hydrodynamic added mass coefficient (${{\rm{C}}}_{{\rm{m}}}$) and a shape parameter (${\rm{\kappa }}$) are 1.85, 1.0 and 3.0, respectively, as used in the previous case study^38^. Another parameter (κ′) controlling the double Gaussian function was estimated to 1.0, assuming that the ratio of minimum and maximum amplitudes is equal to that of deposition and failure thicknesses. This model produced a maximum of 11 m of sea-surface subsidence, maximum of 11 m of sea-surface uplift (Fig. 6a), and the volume of water that changed its level is 26.3 km^3^. The calculated tsunami heights along the Sakishima Island coasts approximated the height of the 1771 tsunami (Fig. 6b,c). The shortage of tsunami height at the western and eastern parts of the calculated area (Fig. 6c) suggests that the model is too simple and subject to improvement based on further detailed bathymetric and structural data. However, our result shows that the proposed large-scale collapse of the accretionary prism is a plausible source of the 1771 tsunami.

## Discussion

Source models for the 1771 Yaeyama tsunamis must be consistent with all evidence, which includes the seafloor geometry, geologic structure, tsunami heights, recurrence interval, distribution of tsunami boulders, seismicity and geodetic measurements.

Previous models^23–25^, which have attributed the tsunami source to active faults and landslides, have assumed a slip greater than 10 m on a fault 70 to 140 km long, a slope failure 8 km wide and a 90 m slide. Because the estimated recurrence interval of giant tsunamis in this region is less than 1000 years^9,11,20^, the slip rate of the fault would have to be approximately 10 m/ky or more, which is greater than that known for any active faults in Japan^42^, suggesting that the slip rate is too high. This highly active fault would create large and clear scarps and deformation on the seafloor, which could be readily identified. Bathymetric maps show a linear scarp of approximately 70 km in length in the eastern part of the forearc basin, which approximately corresponds to the eastern half of the active fault model^25^, but the lineament does not extend west, as does the western part of the fault model (Fig. 1). In addition, seismic profile ECr5 (Fig. 2b), which crosses the area of these fault models^23–25^ shows no evidence of active faulting and folding. Additionally, no large-scale landslide was confirmed on the upper slope. Such a landslide would leave turbidites in the forearc basin, but sediment cores from the forearc basin down slope of the proposed slope failures (Fig. 1) contain no turbidites that can be correlated with the 1771 tsunami^43^.

If the 1771 tsunami was generated by slip on the plate boundary^26^, the entire Ryukyu subduction zone could be the source of the giant tsunamis; however, the distribution of tsunami boulders on other islands in the arc show that giant tsunamis are restricted to the area around Ishigaki-jima and Miyako-jima^21,22^. To explain the distribution of tsunami boulders, the plate boundary model requires strong seismic coupling south of the Sakishima Islands, which is a small part of the 1400-km-long Ryukyu Arc. The reason the strong coupling would be limited to a small part of the shallow plate boundary has not been explained.

Our landslide model is based on seafloor topography and a geologic structure that is specific to this part of the arc, which is consistent with the limited distribution of tsunami boulders^21,22^ and lack of strain accumulation along the plate boundary^12–17^. The scarp heights along the landward margin of the depression exceed 1000 m, indicating that the slide has been repeated; however, the behaviour of the slide mass is not clearly revealed.

**Figure 7:**
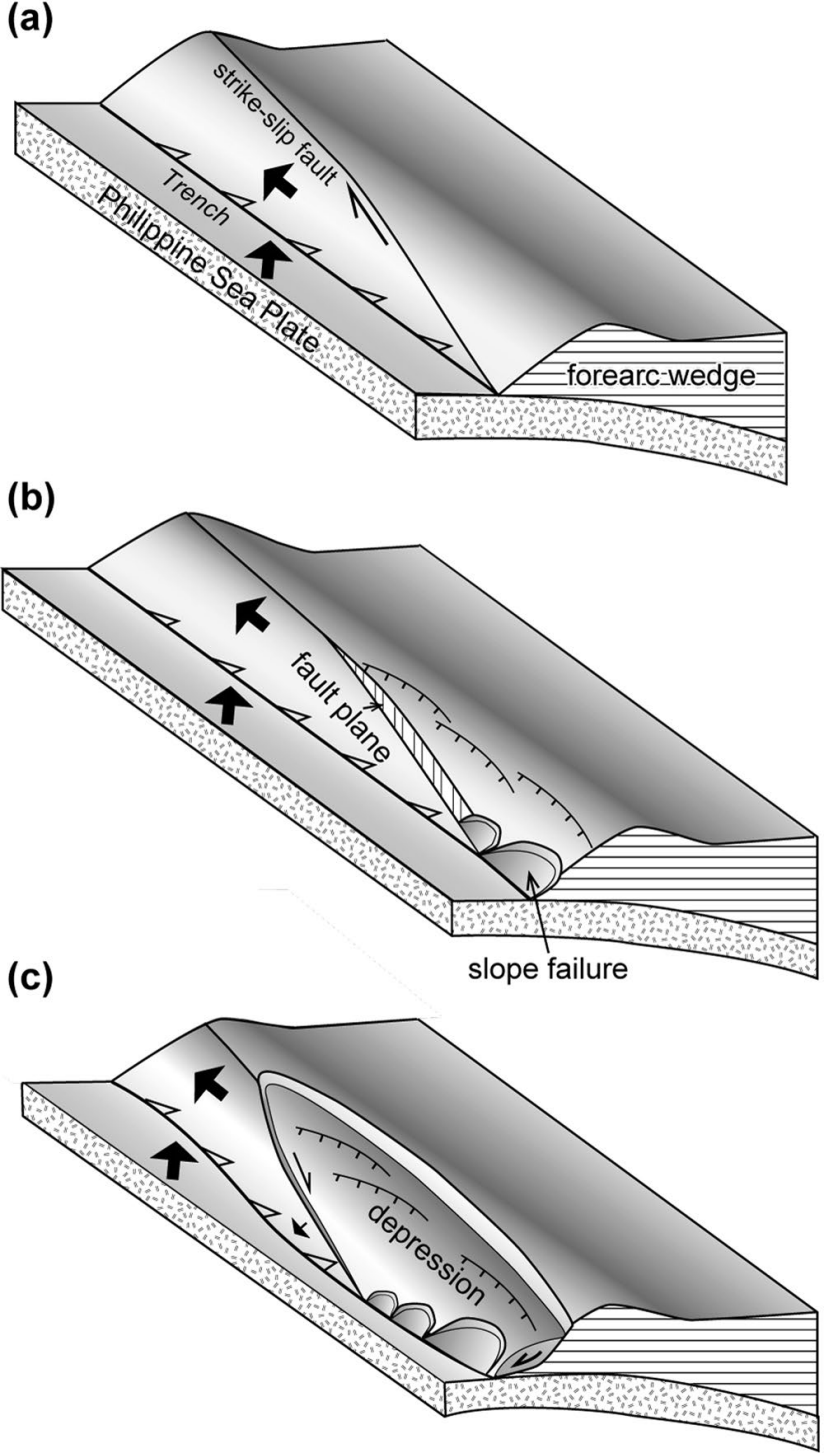
Schematic cartoon showing our hypothesis to explain the mechanism of the accretionary prism collapse due to the lateral dislocation of the accretionary prism seaward block. (**a**) The accretionary prism is obliquely truncated by a strike-slip fault. (**b**) The seaward block of the prism is displaced, and the high-angle fault plane is exposed, which over-steepens the prism and leads to its collapse. (**c**) The collapse extends along the fault scarp as displacement of the seaward block continues, and the landward part of the prism collapses seaward as a large-scale slide mass. The original strike-slip fault is inferred to bend seaward due to the seaward slide of the collapsed prism. Figures were produced using Adobe Illustrator CS6.

We propose a hypothesis that the unusually large-scale collapse within the accretionary prism is caused by strike-slip faulting in the accretionary prism (Fig. 7). Because the azimuth of the fault strike is approximately 95° to the west of 124°E and the trend of the trench changes to 85° east of 124°E (Figs 1 and 3), the eastern fault extension into the depression is inferred to have reached the trench, which obliquely cut the accretionary prism before the sliding began (Fig. 7a). Thus, we infer that a displacement on the strike-slip fault of approximately 40 km has removed a seaward block of the accretionary prism to the west (Fig. 7b,c).

Forearc wedges along subduction zones generally maintain a critical taper wedge, which is described in the Coulomb wedge model^44,45^. If the seaward block of the accretionary prism was removed by strike-slip faulting, the landward portion of the strike-slip fault lost its seaward support and over-steepened, and then, the slope was forced to collapse (Fig. 7c). The collapse probably started as small surface slope failures around the eastern tip of the strike-slip fault (Fig. 7b), and then, the collapse spread landward and westward as the displacement increased (Fig. 7c). The steep slope along the eastern part of seaward margin of the depression (around CDP 66000 in Figs 2b and 5a) was probably the steep scarp formed by strike-slip faulting that obliquely truncated the toe of the accretionary prism. Minor scarps within the depression (Fig. 4) may have been scarps along the landward margin of the depression during the earlier stages of the collapse, and the scarp migrated landward. Although the detailed process of the prism collapse is not known during the gradual displacement of its seaward block, this model is consistent with the fact that the depression is elongated to the ESE, which is oblique to the trench but parallel to the strike-slip fault.

The 1771 tsunami was accompanied by ground shaking that was widely felt in the southern and middle Ryukyu Islands^10,11^, which suggests that an earthquake triggered the collapse event and generated the tsunami. We infer that slow slips and stick slips along the plate boundary landward of the accretionary prism have been pushing the slide mass seaward and destabilized the mass over the period of several hundred years before the 1771 tsunami, and finally, a larger earthquake probably occurred along the plate boundary landward of the depression and triggered the slide.

Our model suggests that strong coupling and large strain accumulation along the Ryukyu Arc are not necessary to explain the 1771 tsunami. Because oblique subduction and development of strike-slip faults on the forearc slope are restricted to the southwestern part of the arc, the probability of giant tsunamis in the middle and northern arc is inferred to be very small, which is supported by the lack of tsunami boulders in those parts of the arc^21,22^. However, Ishigaki-jima and Miyako-jima are at risk for giant tsunamis in the future. Our results also suggest that detailed bathymetric and seismic surveys are necessary to evaluate the potential for giant tsunamis along oblique subduction plate boundaries, regardless of their coupling states.

## Methods

### Bathymetric and seismic data

Bathymetric data for the study area were obtained before 2016 by Japan Coast Guard survey vessels equipped with multi-beam echo sounders mainly along N-S survey lines at intervals of approximately 9 km and 18 km north and south of 23°20′N, respectively. To fill gaps between the survey lines (Supplementary Figure A1), the data were interpolated for the production of a 150-m grid using Generic Mapping Tools^46^.

For the reflection survey, seismic sources included a three-airgun cluster (total volume 17.1 L (1050 inch^3^)) towed at 10 m depth and fired at 50 m intervals at a pressure of 2000 psi (13.8 MPa). The seismic data were recorded using a 240-channel hydrophone streamer, which was 3000 m long and towed at 12 m depth. The data processing sequence included resampling, common midpoint sorting at 6.25 m intervals, datum correction, pre-stack bandpass filtering, amplitude recovery, minimum phase conversion, deconvolution, velocity analysis, multiple suppression, normal moveout correction, mute and 30-fold common midpoint stacking, post-stack time migration, and post-stack bandpass filtering. For the time-to-depth conversion, we used the P-wave velocity model derived from the refraction survey.

For the refraction survey, ocean bottom seismographs were deployed at 5 km intervals to record shots from a non-tuned airgun array (total volume 98.4 L (6000 inch^3^)) fired at 200 m (90 s) intervals. Navigation was provided by the Global Positioning System of the survey vessel and the location of each seismograph was confirmed on the basis of direct wave arrival times^47^. Details of the refraction data processing and velocity analysis are presented in Nishizawa *et al*.^48^.

### Tsunami simulation

To calculate the tsunami propagation from the source to the coast, nonlinear shallow-water equations in spherical coordinates were solved using a finite-difference method^49^. To compute the coastal tsunami heights, we used a grid of bathymetric data with intervals of 6″ (approximately 170 m) resampled from the JTOPO30 30″ grid dataset, which was provided by the Marine Information Research Center, Japan Hydrographic Association. A time step of 0.3 s was used to satisfy the stability condition for the finite-difference method. The computation area extended from 122°45′ to 126°E and from 22°45′ to 25°15′N, with dimensions of 1950 and 1500 grid points in the longitude and latitude directions, respectively. Manning’s roughness coefficient was set to 0.03 m^−1/3^ s, the minimum water depth was clipped to 5 m, and total reflections of tsunami waves from the coastline were assumed.

## Electronic supplementary material

Supplementary Figure 1
