# Supplementary material for: Accretionary prism collapse: a new hypothesis on the source of the 1771 giant tsunami in the Ryukyu Arc, SW Japan
Source: Sci Rep. 2018 Sep 11;8:13620. doi: 10.1038/s41598-018-31956-8 (PMC6134009; doi:10.1038/s41598-018-31956-8)
Supplement: Supplementary file 1 — Supplementary Figure 1 [file 41598_2018_31956_MOESM1_ESM.pdf]

Supplementary Figure

Accretionary prism collapse: a new hypothesis on the source of the 1771 giant tsunami in the Ryukyu Arc, SW Japan

Yukinobu Okamura[1]*, Azusa Nishizawa[2,] Yushiro Fujii[3], Hideaki Yanagisawa[4]

[1] Research Institute of Earthquake and Volcano Geology, Geological Survey of Japan, AIST, 1-1-1 Higashi, Tsukuba, Ibaraki 305-8567, Japan

[2] Hydrographic and Oceanographic Department, Japan Coast Guard, 3-1-1 Kasumigaseki, Chiyoda-ku, Tokyo 100-8932, Japan

[2] National Research Institute for Earth Science and Disaster Resilience, 3-1, Tennodai, Tsukuba, Ibaraki, 305-0006, Japan

[3] International Institute of Seismology and Earthquake Engineering, Building Research Institute, 1 Tachihara, Tsukuba, Ibaraki 305-0802, Japan

[4] Department of Regional Design, Faculty of Liberal Art, Tohoku Gakuin University, 2-1-1 Tenjinzawa, Izumi-ku, Sendai, Miyagi 981-3193, Japan

* Corresponding author: okamura-y@aist.go.jp

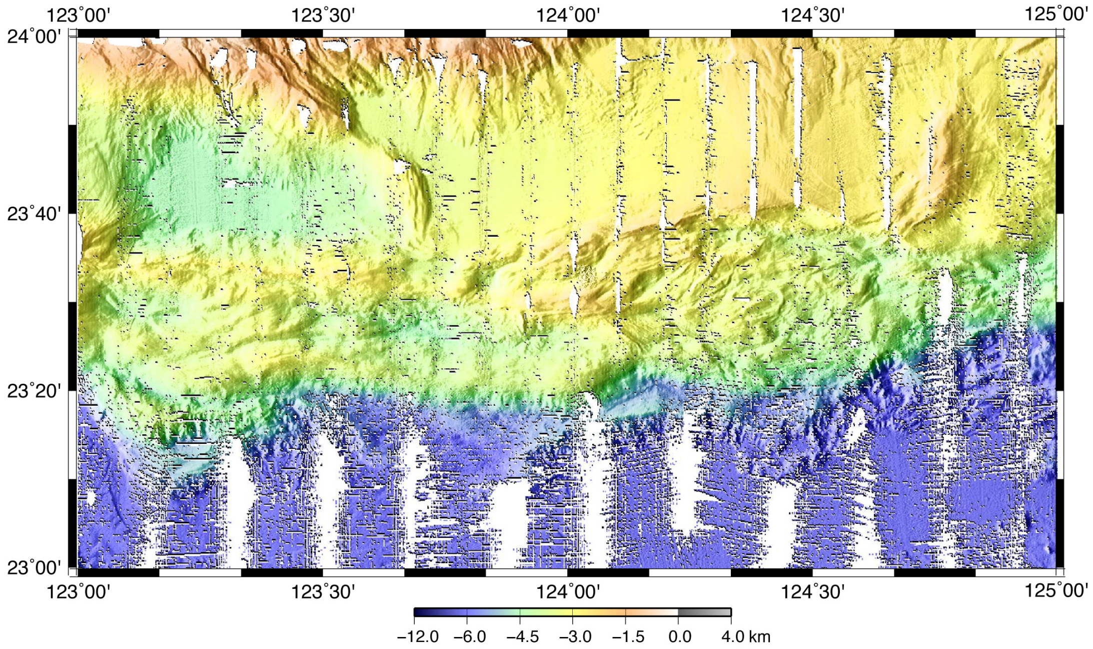

Supplementary Figure A1. Map showing the original bathymetric data.

The data was obtained by multi-narrow-beam echo sounders. White areas are gaps in the data.
